# Computational study of a co-infection model of HIV/AIDS and hepatitis C virus models

**DOI:** 10.1038/s41598-023-48085-6

**Published:** 2023-12-11

**Authors:** Fazal Dayan, Nauman Ahmed, Abdul Bariq, Ali Akgül, Muhammad Jawaz, Muhammad Rafiq, Ali Raza

**Affiliations:** 1 Department of Mathematics, School of Science, University of Management and Technology, Lahore, Pakistan; 2 Department of Mathematics and Statistics, The University of Lahore, Lahore, Pakistan; 3 Department of Mathematics, Art and Science Faculty, Siirt University, 56100 Siirt, Turkey; 4 Near East University, Mathematics Research Center, Department of Mathematics, Near East Boulevard, 99138 Nicosia/Mersin 10, Turkey; 5 Department of Computer Science and Mathematics, Lebanese American University, Beirut, Lebanon; 6 Department of Mathematics, Laghman University, Mehtarlam City, 2701 Laghman Afghanistan; 7 Department of Mathematics, Faculty of Sciences, University of Central Punjab, Lahore, Pakistan; 8 Department of Mathematics, Govt. Maulana Zafar Ali Khan Graduate College Wazirabad, Punjab Higher Education Department (PHED), Lahore, 54000 Pakistan

**Keywords:** Chemical biology, Mathematics and computing

## Abstract

Hepatitis C infection and HIV/AIDS contaminations are normal in certain areas of the world, and because of their geographic overlap, co-infection can’t be precluded as the two illnesses have a similar transmission course. This current work presents a co-infection model of HIV/AIDS and Hepatitis C virus with fuzzy parameters. The application of fuzzy theory aids in tackling the issues associated with measuring uncertainty in the mathematical depiction of diseases. The fuzzy reproduction number and fuzzy equilibrium points have been determined in this context, focusing on a model applicable to a specific group defined by a triangular membership function. Furthermore, for the model, a fuzzy non-standard finite difference (NSFD) technique has been developed, and its convergence is examined within a fuzzy framework. The suggested model is numerically validated, confirming the dependability of the devised NSFD technique, which successfully retains all of the key properties of a continuous dynamical system.

## Introduction

HIV and HCV are microbes that cause huge interruptions and actuation of the immune system. This significant impact on the host’s immune system would make individuals contaminated with HCV more susceptible to HIV and the impacts of the infection. Because of their geographical overlap, there is no question that co-infection with HCV and HIV/AIDS can alter their development, as well as the severity and rate of progression of the disease since every one of these two significantly affects the invulnerable immune system. Increasing the rate of progression of either disease in the presence of the other may play an essential role in increasing the prevalence of the former. Mathematical models have been critical in understanding the spread of infectious diseases that are directly transmissible. Mathematical modeling of infectious disease is a vital tool used by epidemiologists, public health officials, and academics to understand illnesses spread across populations and develop ways for limiting and minimizing their impact. Co-infection refers to disease by at least two different pathogenic creatures. Hepatitis C is a typical co-contamination in individuals living with HIV. A HIV/AIDS and Hepatitis C Virus (HCV) co-infection model is a mathematical model that describes the dynamics of both diseases within a population where individuals can be infected with both HIV and HCV at the same time. Due to the interconnections between the two diseases and the immune response, co-infection models of HIV/AIDS and HCV are complicated. They are an important resource for studying how co-infection influences disease development, transmission dynamics, and treatment effects. Such models can help to guide public health policies and provide methods for efficiently managing and preventing co-infections. Several confection models have been constructed, mathematically examined, and applied^1–8^, just to mention a few. Hepatitis C virus and HIV co-infection, although ineffectively comprehended, is a developing general wellbeing concern, essentially because of their nearby normal pathway relationship. Since the diseases are spread similarly, essentially through needle sharing and sexual action, many individuals are co-contaminated with HIV and HCV. HIV co-infection can expand the sexual and vertical transmission of HCV. HCV-incited liver infection can advance, with 20% to 30% cirrhosis^9^. HCV positivity was related with a 2.6-overlay expanded hazard of AIDS-characterizing ailments^10, 11^. A few hypothetical investigations have analyzed the co-contamination of HIV and different infections like malaria, tuberculosis, and so forth^12–17^. Bhunu and Mushayabasa investigated the co-dynamics of HIV/AIDS and the hepatitis C virus using a deterministic model in order to assess how each disease’s dynamics was influenced by the other while taking treatment effects into account. The findings showed that HCV has a persistent, long-term negative impact on population health, regardless of HIV status, which highlights the need for stronger control strategies in areas with limited resources^18^.

The definitions of susceptibility and infectivity exhibit uncertainty due to the varying degrees of susceptibility and infectivity observed among individuals within the population. Discrepancies can emerge when examining population groups with distinct behaviors, traditions, and age brackets, leading to differences in resistance levels, among other factors. To adequately address these varying individual levels, it is imperative to employ more realistic models. When dealing with epidemic systems related to infectious diseases, a distinct approach is necessary to accommodate these uncertainties. These uncertainties stem from the fact that the strength of an infectious agent’s outbreak relies, among other factors, on the proportions of susceptible and infectious nodes within the network. Given that susceptibility and infectivity inherently encompass vagueness, they serve as ideal concepts for engaging in discussions involving fuzzy logic^19^. As the parameters utilized in epidemic models carry inherent uncertainty, the integration of fuzzy theory becomes a viable approach. The application of fuzzy logic in the realm of biological systems holds significant promise, although it remains relatively underutilized.

An SI model with fuzzy theory was developed by Barros et al., in which the transmission coefficient is addressed as a fuzzy set^20^. They made a comparison between the average count of infected individuals and the mean alteration in virus load, subsequently conducting an analysis of the fundamental reproduction value. Mondal et al. directed their efforts toward plague models, wherein the fuzzy transmission coefficient concept was adopted, leading to the formulation of an SIS model^21^. Renu Verma et al. designed SEIR and SEIRHD models to delineate transmission pathways in the context of the Ebola outbreak. The models integrated fuzzy parameters and explored the existence and stability of equilibria. Significantly, the stability of these equilibrium states was intricately linked to the computation of the basic reproduction number, a task that was facilitated by employing the next-generation matrix^22^. Fuzzy logic was used by Ortega et al. to create predictions in the field of epidemiology, specifically focusing on problems with infectious diseases^23^. A model is developed that focuses on the transition of the HIV-positive population to AIDS, with a particular emphasis on understanding the transmission dynamics from HIV to AIDS. Given the inherent uncertainty of HIV/AIDS, the transmission rate is modeled as a fuzzy set based on viral load^24^. A fuzzy-oriented approach is introduced to epidemiological models concerning the prevalence of HIV within a cohort of individuals engaged in injectable drug use^25^. A comprehensive analysis encompassed various fuzzy scenarios, exploring diverse user counts and different quantities of HIV test samples conducted annually. It’s important to note that these trial sample sizes were tailored to individual cases due to the fluid nature of each community’s evolving environment. Recognizing the evolving nature of communities over time, it’s evident that even the biological parameters utilized within mathematical models are subject to change^26^. Fuzzy models are more insightful than crisp models in this sense. Verma et al. explored a model of Influenza propagation characterized by an asymptotic transmission rate. The rates of disease transmission and mortality were treated as fuzzy sets. Through the utilization of probability measures and fuzzy expected values, they derived the fuzzy basic reproduction number for various subgroups of infected individuals exhibiting different levels of viral loads. Furthermore, a comparative analysis of the basic reproduction numbers between the traditional and fuzzy models was also conducted^27^. Renu et al. constructed a population model using interval values to represent the interrelationships among phytoplankton, zooplankton, and fish populations. This model incorporated a cyrtoid-type functional response^28^.

in the current work, we developed an NSFD techinque to solve a co-infection model of HIV/AIDS and Hepatitis C virus with fuzzy parameters^29^. Employing fuzzy theory aids in addressing the challenges associated with quantifying uncertainty in mathematical modeling of diseases. As a result, the utilization of fuzzy parameters assists in providing a more precise explanation for the transmission of the co-infection model involving HIV/AIDS and Hepatitis C virus.

The novelty of the suggested approach is the creation, application, and assessment of a first-order numerical method in the NSFD conditions. This technique is designed for the co-infection model that represents the behavior of both Hepatitis C infection and HIV/AIDS dynamics, particularly when dealing with fuzzy parameters. The main advantage of this study is the introduction of fuzzy parameters into the HIV/AIDS coinfection model. Unlike traditional models, which frequently assume precise parameter values, the inclusion of fuzzy parameters allows for a more nuanced consideration of the uncertainty and imprecision inherent in real-world circumstances. The model can more accurately depict the complexities and variances prevalent in real-world systems because to this increased level of realism. The current study provides a more detailed depiction of the intricate connections between HIV and AIDS dynamics by using fuzzy parameters for uncertain or ambiguous parameter values. This research is structured as follows. “A co-infection model of HIV/AIDS and hepatitis C virus with fuzzy parameters” section presents some basic concepts that will be employed in this study, as well as a discussion of the development of a co-infection model of HIV/AIDS and HCV virus with fuzzy parameters. In “Mathematical analysis” section, we discussed the fuzzy reproduction number and fuzzy equilibrium analysis. “Numerical modeling” section contains the presentation of numerical solution and simulation outcomes. “Conclusion” section summarizes the research’s concluding remarks and future directions.

## A co-infection model of HIV/AIDS and hepatitis C virus with fuzzy parameters

In this section, we present an extended co-infection model involving HIV/AIDS and Hepatitis C viruses, where the incorporation of fuzzy parameters is considered. To initiate, we outline some fundamental definitions that will serve as a foundation for this study.

### Fuzzy subset^30^

The membership function $\mu _S(u): U rightarrow [0, 1]$ denotes a fuzzy subset *S* within the universe set *U*, where $\mu _S(u)$ represents the degree of membership of *u* in the fuzzy set *S*.

### Triangular fuzzy number (TFN)^30^

The triplet $A=(a, b, c)$ qualifies as a TFN when its membership function is characterized by

1 $$\begin{aligned} \mu _A(x)=\left\{ \begin{array}{ll} 0, & \quad x\le a\\ \frac{x-a}{b-a}, & \quad a < x\le b \\ \frac{x-c}{b-r}, & \quad b < x\le c \\ 0, & \quad c\le x\\ \end{array}\right. \end{aligned}$$

with *a* being less than or equal to *b*, and *b* being less than or equal to *c*.

### Expected value (EV) of a TFN^31^

The EV of a TFN is given by

2 $$\begin{aligned} E[A]=\frac{a+2b+c}{4} \end{aligned}$$

### Fuzzy basic reproductive number (BRN)^31^

The fuzzy BRN of a TFN $R_0 (\nu )$ is given by

3 $$\begin{aligned} R_0^f=E[R_0 (\nu )] \end{aligned}$$

Consider the model that has been talked about by Bhunu and Mushayabasa^18^.

4 $$\begin{aligned} {\frac{dS}{dt}}& = {\Lambda -(\mu +\lambda _h+\lambda _c)S+r_1I_c} \end{aligned}$$

5 $$\begin{aligned} {\frac{dI_c}{dt}}& = {\lambda _cS-(\mu +d_c+r_1+\delta \lambda _h)I_c} \end{aligned}$$

6 $$\begin{aligned} {\frac{dI_h}{dt}}& = {\lambda _hS+r_2I_{hc}-(\mu +\rho _1+\sigma _1\lambda _c)I_h} \end{aligned}$$

7 $$\begin{aligned} {\frac{dA_h}{dt}}& = {\rho _1I_h+r_3A_{hc}-(\mu +d_a+\theta _1+\sigma _2\lambda _c)A_h} \end{aligned}$$

8 $$\begin{aligned} {\frac{dA_t}{dt}}& = {\theta _1A_{h}+r_4A_{tc}-(\mu +d_a+\sigma _3\lambda _c)A_t} \end{aligned}$$

9 $$\begin{aligned} {\frac{dI_{hc}}{dt}}& = {\delta _{hc}I_c+\sigma _1\lambda _cI_h-(\mu +d_c+r_2+\rho _2)I_{hc}} \end{aligned}$$

10 $$\begin{aligned} {\frac{dA_{hc}}{dt}}& = {\rho _2 I_{hc}+\sigma _2\lambda _cA_h-(\mu +\theta _2+d_a+d_c+r_3)A_{hc}} \end{aligned}$$

11 $$\begin{aligned} {\frac{dA_{tc}}{dt}}& = {\theta _2 A_{hc}+\sigma _3\lambda _cA_t-(\mu +r_4+d_a+d_c)A_{tc}} \end{aligned}$$

**Figure 1:**
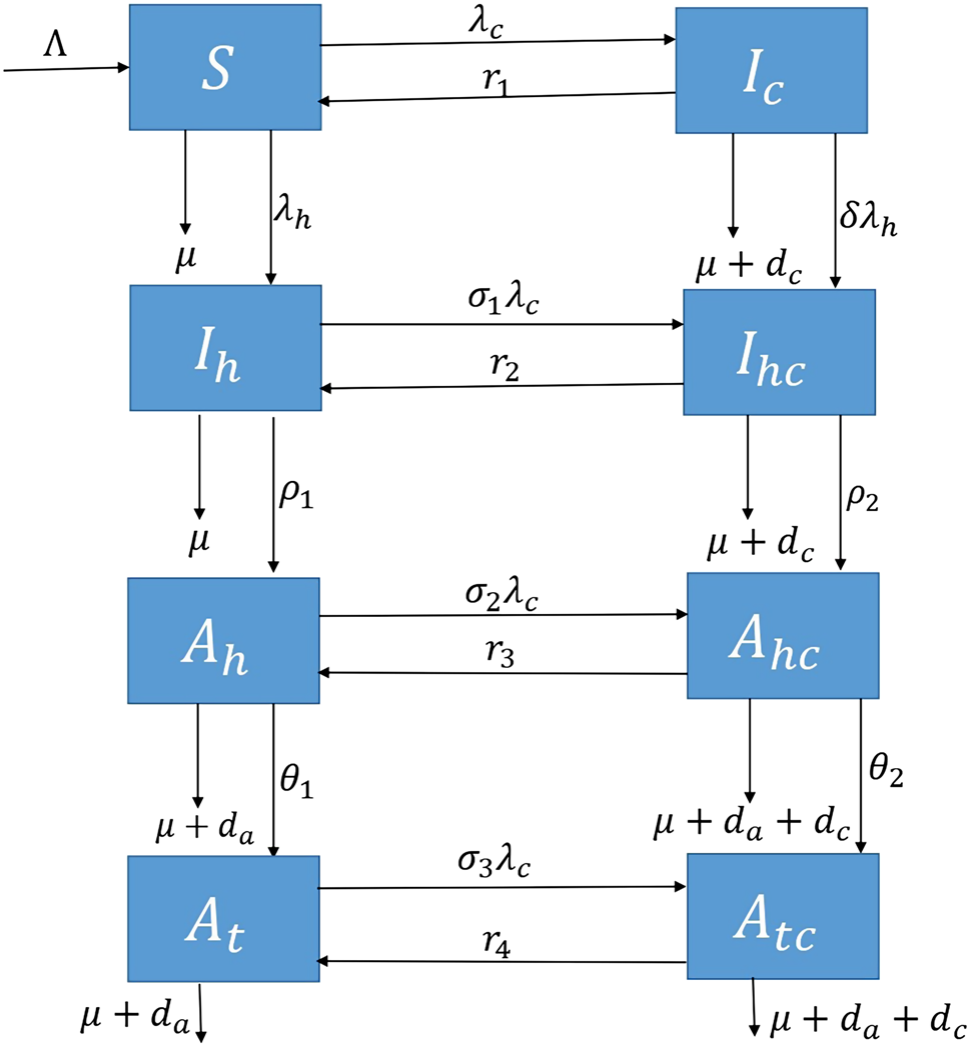
Flowchart of the model.

The flowchart of the studied model is shown in Fig. 1.

The fuzzy model corresponding to the above model can be expressed as

12 $$\begin{aligned} {\frac{dS}{dt}}& = {\Lambda -(\mu +\lambda _h+\lambda _c)S+r_1I_c} \end{aligned}$$

13 $$\begin{aligned} {\frac{dI_c}{dt}}& = {\lambda _cS-(\mu +d_c(\nu )+r_1+\delta \lambda _h)I_c} \end{aligned}$$

14 $$\begin{aligned} {\frac{dI_h}{dt}}& = {\lambda _hS+r_2I_{hc}-(\mu +\rho _1+\sigma _1\lambda _c)I_h} \end{aligned}$$

15 $$\begin{aligned} {\frac{dA_h}{dt}}& = {\rho _1I_h+r_3A_{hc}-(\mu +d_a(\nu )+\theta _1+\sigma _2\lambda _c)A_h} \end{aligned}$$

16 $$\begin{aligned} {\frac{dA_t}{dt}}& = {\theta _1A_{h}+r_4A_{tc}-(\mu +d_a(\nu )+\sigma _3\lambda _c)A_t} \end{aligned}$$

17 $$\begin{aligned} {\frac{dI_{hc}}{dt}}& = {\delta _{hc}I_c+\sigma _1\lambda _cI_h-(\mu +d_c(\nu )+r_2+\rho _2)I_{hc}} \end{aligned}$$

18 $$\begin{aligned} {\frac{dA_{hc}}{dt}}& = {\rho _2 I_{hc}+\sigma _2\lambda _cA_h-(\mu +\theta _2+d_a(\nu )+d_c(\nu )+r_3)A_{hc}} \end{aligned}$$

19 $$\begin{aligned} {\frac{dA_{tc}}{dt}}& = {\theta _2 A_{hc}+\sigma _3\lambda _cA_t-(\mu +r_4+d_a(\nu )+d_c(\nu ))A_{tc}} \end{aligned}$$

When developing the membership function, we assumed that when the number of virus-loads in an individual is low, the possibility of transmission becomes low. Furthermore, there is a minimum virus-load threshold required for any potential transmission to occur. Furthermore, there is a specific amount of virus-load at which the transmission rate peaks and equals one. We assume that the contact transmission rate $\beta _c(\nu )$, $\beta _h(\nu )$, the mortality rates $d_c(\nu )$ and $d_a(\nu )$ due to HCV and AIDS respectively are fuzzy numbers that rely on the viral load of each individual in the population. Let $\beta _c(\nu )$ is the product of the effective contact rate for HCV infection and the chance of its transmission per contact and can be defined as

20 $$\begin{aligned} \beta _c(\nu )=\left\{ \begin{array}{ll} 0, & \quad \nu<\nu _m \\ \frac{\nu -\nu _m}{\nu _0-\nu _m}, & \quad \nu _m\le \nu \le \nu _0 \\ 1, & \quad \nu _0<\nu <\nu _M\\ \end{array}\right. \end{aligned}$$

$\beta _h(\nu )$ is the product of the effective contact rate for HIV infection and chance of its transmission per contact and can be defined as

21 $$\begin{aligned} \beta _h(\nu )=\left\{ \begin{array}{ll} 0, & \quad \nu<\nu _m \\ \frac{\nu -\nu _m}{\nu _0-\nu _m}, & \quad \nu _m\le \nu \le \nu _0 \\ 1, & \quad \nu _0<\nu <\nu _M\\ \end{array}\right. \end{aligned}$$

The mortality rates $d_c (\nu )$ for HCV and $d_a (\nu )$ for AIDS can also be treated as fuzzy numbers since these rates rise with the progression of disease infection. They can be defined as

22 $$\begin{aligned} d_c(\nu )=\left\{ \begin{array}{ll} \frac{(1-\zeta )-\epsilon _0}{\nu _m}, & \quad 0\le \nu \le \nu _m \\ 1-\zeta , & \quad \nu _m<\nu \\ \end{array}\right. \end{aligned}$$

and

23 $$\begin{aligned} d_a(\nu )=\left\{ \begin{array}{ll} \frac{(1-\xi )-\epsilon _0}{\nu _m}, & \quad 0\le \nu \le \nu _m \\ 1-\xi , & \quad \nu _m<\nu \\ \end{array}\right. \end{aligned}$$

The mortality rates $d_c (\nu )$ attributed to HCV and $d_a (\nu )$ associated with AIDS exhibit higher values when the viral load $\nu$ reaches its peak and the maximum deaths are 1 − $\zeta$, $(\zeta \ge 0)$ and 1 − $\xi$, $(\xi \ge 0)$.

Details of the other parameters and variables used in our model is given below:

- *S* :  Susceptible
- $I_h:$ HIV positive-only individuals not yet showing AIDS symtoms
- $I_c:$ People infected only with hepatitis C
- $A_h:$ AIDS patients not yet on antiretroviral therapy (AT)
- $A_t:$ AIDS patientst on AT
- $I_{hc}:$ HIV positive who does not yet have symptoms of AIDS with dual HCV infection
- $A_{hc}:$ AIDS patients with dual HCV infection not on AT
- $A_{tc}:$ AIDS patients with dual HCV infection on AT
- $\Lambda :$ Constant birth rate
- $\mu :$ Natural death rate
- $\lambda _h:$ Force of infection associated with HIV infection, where $\lambda _h=\frac{\beta _h(\nu )[I_h+\varphi I_{hc}]}{N}$
- $\lambda _c:$ Force of infection associated with HIV infection, where $\lambda _c=\frac{\beta _c(\nu )[I_h+\eta I_{hc}]}{N}$
- $r_1:$ Rate at which HCV infected individuals in $I_c$ move back into the class of the susceptible
- $r_2:$ Rate at which dually infected people in class $I_{hc}$ are treated for HCV to move back into class $I_h$
- $r_3:$ Rate at which dually infected people in class $A_{hc}$ are treated for HCV to move back into class $A_h$
- $r_4:$ Rate at which dually infected people in class $A_{tc}$ are treated for HCV to move back into class $A_t$
- $\rho _1:$ Rate at which susceptibles infected with HIV enters class $I_h$ and progress to $A_h$
- $\rho _2:$ Rate at which people in class $I_{hc}$ progress to $A_{hc}$
- $\theta _1:$ Rate at which individuals in stage $A_h$ detected and put on treatment to enter the class $A_t$
- $\theta _2:$ Rate at which AIDS patients dually infected with HCV in class $A_{hc}$ are detected and put on antiretroviral therapy to get into class $A_{tc}$
- $\sigma \lambda _c:$ Rate at which individuals in class $I_h$ are infected with HCV to enter $I_{hc}$
- $\delta \lambda _h:$ Rate at which HCV only infected individuals in class $I_c$ are infected with HIV to move into class $I_{hc}$.

## Mathematical analysis

### HCV-only sub model

For the HCV Sub model, $I_h=A_h=A_t= I_{hc}=A_{hc}= A_{tc}=0$, So the system of Eqs. (12–19) reduces to

24 $$\begin{aligned} {\frac{dS}{dt}}& = {\Lambda -(\mu +\lambda _h+\lambda _c)S+r_1I_c} \end{aligned}$$

25 $$\begin{aligned} {\frac{dI_c}{dt}}& = {\lambda _cS-(\mu +d_c(\nu )+r_1+\delta \lambda _h)I_c} \end{aligned}$$

#### Fuzzy equilibrium analysis

This model has a virus free equilibrium point (VFE) and two endemic equilibrium (EE) points.

- **Case 1.** If $\nu <\nu _m$, then $\beta _c(\nu )=0$ and $\lambda _c=0$. Substituting it the in Eq. (25), we get $I_c=0$ and from Eq. (24) we get $S=\frac{\Lambda }{\mu }$. Therefore, we obtain:
  $$\begin{aligned} E_c^0=\left( \frac{\Lambda }{\mu }, 0, 0, 0, 0, 0, 0, 0\right) . \end{aligned}$$
  This scenario is known as the VFE point, describes a situation in which the hepatitis C virus is not present in the population. From a biological perspective, the disease is considered eradicated when the virus level in the population falls below the threshold required for disease transmission.
- **Case 2.** If $\nu _m\le \nu \le \nu _0$, then $\beta _c(\nu )=\frac{\nu -\nu _m}{\nu _0-\nu _m}$ and from Eq. (25), we have
  26 $$\begin{aligned} {\lambda _cS-(\mu +d_c(\nu )+r_1)I_c}=0 \end{aligned}$$
  By putting $\lambda _c=\frac{\beta _c(\nu )I_c}{N}$ in Eq. (26) it becomes
  $$\begin{aligned}{} & {} \frac{\beta _c(\nu )I_c}{N}S-(\mu +d_c(\nu )+r_1)I_c=0\\{} & {} I_c\left[ \frac{\beta _c(\nu )}{N}S-(\mu +d_c(\nu )+r_1)\right] =0 \end{aligned}$$
  since $$I_c\ne 0$$, $$\Rightarrow \frac{\beta _c(\nu )}{N}S-(\mu +d_c(\nu )+r_1)=0$$
  27 $$\begin{aligned} \Rightarrow I_c=(R_c^*-1)S \end{aligned}$$
  We put the value of $I_c$ in Eq. (24) and obtained
  28 $$\begin{aligned} S=\frac{\Lambda }{\beta _c(\nu )(1-\frac{1}{R_c^*})+\mu -r_1(R_c^*-1)}. \end{aligned}$$
  We achieve the point of equilibrium point
  $$\begin{aligned} E_c^*=(S^*, I_c^*, 0, 0, 0, 0, 0, 0) \end{aligned}$$
  where $$S^*=\frac{\Lambda }{\beta _c(\nu )(1-\frac{1}{R_c^*})+\mu -r_1(R_c^*-1)}$$ and $$I_c^*=(R_c^*-1)S$$.
- **Case 3.** If $\nu _0<\nu <\nu _M$, then $\beta (\nu )=1$ and we obtain
  $$\begin{aligned} E_c^{**}=(S^{**}, I_c^{**}, 0, 0, 0, 0, 0, 0) \end{aligned}$$
  where $$S^{**}=\frac{\Lambda }{(1-\frac{1}{R_c^{**}})+\mu -r_1(R_c^{**}-1)}$$ and $$I_c^{**}=(R_c^{**}-1)S$$.

#### Fuzzy BRN

The BRN of the HCV only sub model is given by

29 $$\begin{aligned} {R_c=\frac{\beta _c(\nu )}{\mu +d_c(\nu )+r_1}} \end{aligned}$$

As $R_c$ depends on the viral load $\nu$, we examine it across various levels of viral quantities as follows:

- **Case 1.** If $\nu <\nu _m$, then $\beta _c(\nu )=0$ and $R_c(\nu )=0$.
- **Case 2.** If $\nu _m\le \nu \le \nu _0$, then $\beta _c(\nu )=\frac{\nu -\nu _m}{\nu _0-\nu _m}$ and $R_c(\nu )=\frac{\beta _c(\nu )}{\mu +d_c(\nu )+r_1}$.
- **Case 3.** If $\nu _0<\nu <\nu _M$, then $\beta _c(\nu )=1$ and $R_c(\nu )=\frac{1}{\mu +d_c(\nu )+r_1}$. The relationship between the BRN $R_c(\nu )$, and the viral load, indicated as $\nu$, is such that $R_c(\nu )$ rises as the viral burden rises. This function is specifically defined as a fuzzy variable. As a result, the expected value of $R_c(\nu )$ is similarly well defined, allowing it to be represented as a TFN as
  30 $$\begin{aligned} R_c(\nu )=\left( 0, \frac{\beta _c(\nu )}{\mu +d_c(\nu )+r_1}, \frac{1}{\mu +d_c(\nu )+r_1}\right) \end{aligned}$$
  Now by using Eqs. (2) and (3), we find the fuzzy BRN as follows:
  31 $$\begin{aligned}{} & {} R_c^f=E[R_c (\nu )] \end{aligned}$$
  32 $$\begin{aligned}{} & {} R_c^f=\frac{2\beta _c(\nu )+1}{4(\mu +d_c(\nu )+r_1)} \end{aligned}$$

#### Stability analysis of equilibria

Suppose

33 $$\begin{aligned} A_1& = {\Lambda -(\mu +\lambda _h+\lambda _c)S+r_1I_c} \end{aligned}$$

34 $$\begin{aligned} A_2& = {\lambda _cS-(\mu +d_c(\nu )+r_1+\delta \lambda _h)I_c} \end{aligned}$$

The Jacobean of the system (33–34) is

$$\begin{aligned} J=\left[ \begin{array}{cc} -\beta _c(\nu ) \left[ \frac{I_c^2}{(S+I_c)^2}\right] -\mu &{} -\beta _c(\nu ) \left[ \frac{S^2}{(S+I_c)^2}\right] +r_1 \\ \beta _c(\nu ) \left[ \frac{I_c^2}{(S+I_c)^2}\right] &{} \beta _c(\nu ) \left[ \frac{S^2}{(S+I_c)^2}\right] -(\mu +d_c(\nu )+r_1) \\ \end{array} \right] =0 \end{aligned}$$

The Jacobean of the system (33–34) at VFE point is

$$\begin{aligned} J(S^0,I_c^0 )=\left[ \begin{array}{cc} -\mu &{} r_1 \\ 0 &{} -(\mu +d_c(\nu )+r_1) \\ \end{array} \right] =0 \end{aligned}$$

The steady-state will be locally asymptotically stable iff absolute eigenvalues of the above Jacobean matrices are less than the unity i.e $|\lambda _i |<1$, $i=1,2$. From the above Jacobean matrix we obtain the eigenvalue $\lambda _1=-\mu <1$ and $\lambda _2=-(\mu +d_c(\nu )+r_1)<1$. Since all eigenvalues are smaller than unity, this verifies the intended outcome

Now we study the stability of EE points $$E_c^*$$ and $$E_c^{**}$$ respectively. The Jacobean of the system (33–34) at EE point $$E_c^*$$ is

$$\begin{aligned} J(S^*,I_c^* )=\left[ \begin{array}{cc} -\beta _c(\nu ) \left( 1-\frac{1}{R_c}\right) ^2-\mu &{} r_1-\frac{\beta _c(\nu )}{R_c^2} \\ \beta _c(\nu ) \left( 1-\frac{1}{R_c}\right) ^2 &{} \frac{\beta _c(\nu )}{R_c^2}-(\mu +d_c(\nu )+r_1) \\ \end{array} \right] =0 \end{aligned}$$

where $$R_c(\nu )=\frac{\beta _c(\nu )}{\mu +d_c(\nu )+r_1}$$.

$$\begin{aligned}{} & {} trace[J(S^*, I_c^* )]=-\beta _c(\nu ) \left( 1-\frac{2}{R_c}\right) ^2-(2\mu +d_c(\nu )+r_1)<0\\{} & {} \det [J(S^*, I_c^* )]=\mu (\mu +d_c(\nu )+r_1)\left( (1-\frac{1}{R_c})(1+\frac{(\mu +d_c(\nu ))R_c}{\mu }(1-\frac{1}{R_c})\right) >0 \end{aligned}$$

Due to the negative trace and positive determinant, the steady state $E_c^*$ can be confirmed as locally asymptotically stable.

The Jacobean of the system (33–34) at EE point $$E_c^{**}$$ is

$$\begin{aligned} J(S^{**},I_c^{**} )=\left[ \begin{array}{cc} - \left( 1-\frac{1}{R_c}\right) ^2-\mu &{} r_1-\frac{1}{R_c^2} \\ \left( 1-\frac{1}{R_c}\right) ^2 &{} \frac{1}{R_c^2}-(\mu +d_c(\nu )+r_1) \\ \end{array} \right] =0 \end{aligned}$$

where $$R_c(\nu )=\frac{1}{\mu +d_c(\nu )+r_1}$$.

Again, the trace of the above matrix is negative and the determinant is positive, hence the steady state $$E_c^{**}$$ is locally asymptotically stable.

### HIV-only sub model

For the HIV sub model, $I_c= I_{hc}=A_{hc}= A_{tc}=0$ so the system of equations (12–19) reduces to

35 $$\begin{aligned} {\frac{dS}{dt}}& = {\Lambda -(\mu +\lambda _h)S} \end{aligned}$$

36 $$\begin{aligned} {\frac{dI_h}{dt}}& = {\lambda _hS-(\mu +\rho _1)I_h} \end{aligned}$$

37 $$\begin{aligned} {\frac{dA_h}{dt}}& = {\rho _1I_h-(\mu +d_a(\nu )+\theta _1)A_h} \end{aligned}$$

38 $$\begin{aligned} {\frac{dA_t}{dt}}& = {\theta _1A_{h}-(\mu +d_a(\nu ))A_t} \end{aligned}$$

#### Fuzzy equilibrium analysis

This sub model has a VFE point and two EE points.

- **Case 1.** If $\nu <\nu _m$, then $\beta _h(\nu )=0$ and $\lambda _h=0$. In this case, we obtain:
  $$\begin{aligned} E_h^0=\left( \frac{\Lambda }{\mu }, 0, 0, 0, 0, 0, 0, 0\right) . \end{aligned}$$
  This is the VFE point, signifying that HIV is not present in the population. The disease is deemed eliminated biologically when the viral level falls below the minimum threshold required for disease transmission within the population.
- **Case 2.** If $\nu _m\le \nu \le \nu _0$, then $\beta _c(\nu )=\frac{\nu -\nu _m}{\nu _0-\nu _m}$ and we get
  $$\begin{aligned} E_h^*=(S^*, 0, I_h^*, A_h^*, A_t^*, 0, 0, 0), \end{aligned}$$
  where
  $$\begin{aligned} S^*& = \frac{\Lambda (\mu +d_a(\nu )+\rho _1)}{\left[ \mu (\mu +d_a(\nu )+\rho _1)+(\mu +\rho _1)(\mu +d_a(\nu ))(R_h-1)\right] }\\ I_h^*& = \frac{\Lambda (\mu +d_a(\nu ))(R_h-1)}{\left[ \mu (\mu +d_a(\nu )+\rho _1)+(\mu +\rho _1)(\mu +d_a(\nu ))(R_h-1)\right] }\\ A_h^*& = \frac{\Lambda \rho _1(\mu +d_a(\nu ))(R_h-1)}{(\mu +d_a(\nu )+\theta _1)[\mu (\mu +d_a(\nu )+\rho _1)+(\mu +\rho _1)(\mu +d_a(\nu ))](R_h-1)} \\ A_t^*& = \frac{\Lambda \theta _1\rho _1(R_h-1)}{(\mu +d_a(\nu )+\theta _1)[\mu (\mu +d_a(\nu )+\rho _1)+(\mu +\rho _1)(\mu +d_a(\nu ))](R_h-1)} \end{aligned}$$
- **Case 3.** If $\nu _0<\nu <\nu _M$, then $\beta (\nu )=1$ and we obtain
  $$\begin{aligned} E_c^{**}=(S^{**},0,I_h^{**},A_h^{**},A_t^{**},0,0,0), \end{aligned}$$
  where
  $$\begin{aligned} S^{**}& = \frac{\Lambda (\mu +d_a(\nu )+\rho _1)}{\left[ \mu (\mu +d_a(\nu )+\rho _1)+(\mu +\rho _1)(\mu +d_a(\nu ))(R_h-1)\right] }\\ I_h^{**}& = \frac{\Lambda (\mu +d_a(\nu ))(R_h-1)}{\left[ \mu (\mu +d_a(\nu )+\rho _1)+(\mu +\rho _1)(\mu +d_a(\nu ))(R_h-1)\right] }\\ A_h^{**}& = \frac{\Lambda \rho _1(\mu +d_a(\nu ))(R_h-1)}{(\mu +d_a(\nu )+\theta _1)[\mu (\mu +d_a(\nu )+\rho _1)+(\mu +\rho _1)(\mu +d_a(\nu ))](R_h-1)} \\ A_t^{**}& = \frac{\Lambda \theta _1\rho _1(R_h-1)}{(\mu +d_a(\nu )+\theta _1)[\mu (\mu +d_a(\nu )+\rho _1)+(\mu +\rho _1)(\mu +d_a(\nu ))](R_h-1)} \end{aligned}$$

The equilibrium points $$E_h^*$$ and $$E_h^{**}$$ are referred to as EE points. These points arise when the virus surpasses the minimum threshold and continues to exist within the population.

#### Fuzzy BRN

We utilize the approach of the next generation matrix technique to compute the reproduction number, which is expressed as

39 $$\begin{aligned} {R_h=\frac{\beta _h(\nu )}{\mu +\rho _1}} \end{aligned}$$

Since $R_h$ depends on the quantity of virus $\nu$, we study it for various virus quantities.

- **Case 1.** If $\nu <\nu _m$, then $\beta _h(\nu )=0$ and $R_h(\nu )=0$.
- **Case 2.** If $\nu _m\le \nu \le \nu _0$, then $\beta _h(\nu )=\frac{\nu -\nu _m}{\nu _0-\nu _m}$ and $R_h(\nu )=\frac{\beta _h(\nu )}{\mu +\rho _1}$.
- **Case 3.** If $\nu _0<\nu <\nu _M$, then $\beta _h(\nu )=1$ and $R_h(\nu )=\frac{1}{\mu +\rho _1}$. The disease-dependent function $R_h(\nu )$ correlates positively with the disease parameter $\nu$, and its definition includes a fuzzy variable. As a result, the EV of $R_h(\nu )$ is well defined, and its representation can be written as a TFN, as follows:
  40 $$\begin{aligned} R_h(\nu )=\left( 0, \frac{\beta _h(\nu )}{\mu +\rho _1}, \frac{1}{\mu +\rho _1}\right) \end{aligned}$$
  Now by using Eqs. (2) and (3), we find the fuzzy BRN as follows:
  41 $$\begin{aligned} R_h^f& = E[R_h (\nu )] \end{aligned}$$
  42 $$\begin{aligned} R_h^f& = \frac{2\beta _h(\nu )+1}{4\mu +\rho _1} \end{aligned}$$

#### Stability analysis of equilibria

Suppose

43 $$\begin{aligned} A_3& = {\Lambda -(\mu +\lambda _h)S} \end{aligned}$$

44 $$\begin{aligned} A_4& = {\lambda _hS-(\mu +\rho _1)I_h} \end{aligned}$$

45 $$\begin{aligned} A_5& = {\rho _1I_h-(\mu +d_a(\nu )+\theta _1)A_h} \end{aligned}$$

46 $$\begin{aligned} A_6& = {\theta _1A_{h}-(\mu +d_a(\nu ))A_t} \end{aligned}$$

Jacobean of the system (43)–(46) at VFE is

$$\begin{aligned} J(S^0,I_h^0,A_h^0,A_t^0)=\left[ \begin{array}{cccc} -\mu &{} 0 &{} 0 &{} 0 \\ 0 &{} -(\mu +\rho _1)&{} 0 &{} 0 \\ 0 &{} \rho _1&{} -(\mu +d_a(\nu )+\theta _1) &{} 0 \\ 0 &{} 0&{} -\theta _1 &{} -(\mu +d_a(\nu )) \\ \end{array} \right] =0 \end{aligned}$$

Here $\lambda _1=-\mu <1$, $\lambda _2=-(\mu +\rho _1)<1$, $\lambda _3=-(\mu +d_a(\nu )+\theta _1)<1$ and $\lambda _4=-(\mu +d_a(\nu ))<1$. Since all eigenvalues are smaller than unity, this verifies the intended outcome

Now we study the stability of the EE points $$E_h^*$$ and $$E_h^{**}$$ respectively. The Jacobean of the system (43–46) at the EE $$E_h^*$$ is

$$\begin{aligned} J(E_h^*)=\left[ \begin{array}{cccc} \frac{\partial A_3}{\partial S}(E_h^*) &{} \frac{\partial A_3}{\partial I_h}(E_h^*) &{} \frac{\partial A_3}{\partial A_h}(E_h^*) &{} \frac{\partial A_3}{\partial A_t}(E_h^*) \\ \frac{\partial A_4}{\partial S}(E_h^*) &{} \frac{\partial A_4}{\partial I_h}(E_h^*) &{} \frac{\partial A_4}{\partial A_h}(E_h^*) &{} \frac{\partial A_4}{\partial A_t}(E_h^*) \\ \frac{\partial A_5}{\partial S}(E_h^*) &{} \frac{\partial A_5}{\partial I_h}(E_h^*) &{} \frac{\partial A_5}{\partial A_h}(E_h^*) &{} \frac{\partial A_5}{\partial A_t}(E_h^*) \\ \frac{\partial A_6}{\partial S}(E_h^*) &{} \frac{\partial A_6}{\partial I_h}(E_h^*) &{} \frac{\partial A_6}{\partial A_h}(E_h^*) &{} \frac{\partial A_6}{\partial A_t}(E_h^*) \\ \end{array} \right] \end{aligned}$$

Since the algebraic form of the solution of the Eigenvalues of the above Jacobean matrix is quite complicated, therefore we calculate it numerically. Here $\lambda _1=-0.3114 + 0.2909i$, $\lambda _2=-0.3114 - 0.2909i$, $\lambda _3=-1.0649$ and $\lambda _4=-0.8330$. Since all the eigenvalues of EE point $E_h^*$ are negative, therefore, the EE point $E_h^*$ is locally asymptotically stable.

The Jacobean of the system (43–46) at EE $$E_h^{**}$$ is

$$\begin{aligned} J(E_h^{**})=\left[ \begin{array}{cccc} \frac{\partial A_3}{\partial S}(E_h^{**}) &{} \frac{\partial A_3}{\partial I_h}(E_h^{**}) &{} \frac{\partial A_3}{\partial A_h}(E_h^{**}) &{} \frac{\partial A_3}{\partial A_t}(E_h^{**}) \\ \frac{\partial A_4}{\partial S}(E_h^{**}) &{} \frac{\partial A_4}{\partial I_h}(E_h^{**}) &{} \frac{\partial A_4}{\partial A_h}(E_h^{**}) &{} \frac{\partial A_4}{\partial A_t}(E_h^{**}) \\ \frac{\partial A_5}{\partial S}(E_h^{**}) &{} \frac{\partial A_5}{\partial I_h}(E_h^{**}) &{} \frac{\partial A_5}{\partial A_h}(E_h^{**}) &{} \frac{\partial A_5}{\partial A_t}(E_h^{**}) \\ \frac{\partial A_6}{\partial S}(E_h^{**}) &{} \frac{\partial A_6}{\partial I_h}(E_h^{**}) &{} \frac{\partial A_6}{\partial A_h}(E_h^{**}) &{} \frac{\partial A_6}{\partial A_t}(E_h^{**}) \\ \end{array} \right] \end{aligned}$$

Again, we calculate the eigenvalues numerically which are given as: $$\lambda _1=-0.2338 + 0.2543i$$, $$\lambda _2=-0.2338 - 0.2543i$$, $$\lambda _3=-0.8330$$ and $$\lambda _4=-0.5761$$. This proves that the EE point $$E_h^{**}$$ is also locally asymptotically stable.

## Numerical modeling

In this section, we will investigate a novel approach, the NSFD technique, which relies on Micken’s theory for the solutions of the dynamic systems (24, 25) and (35–38).

### NSFD scheme for HCV only sub model

NSFD scheme for the system (24, 25) is

47 $$\begin{aligned} S^{n+1}& = \frac{(S^n+\Lambda h+hr_1I_c^n)(S^n+I_c^n)}{S^n+I_c^n+h(\beta _c(\nu )I_c^n+\mu (S^n+I_c^n))} \end{aligned}$$

48 $$\begin{aligned} {I_c^{n+1}}& = \frac{I_c^n (S^n+I_c^n )+\beta _c(\nu )I_c^n S^n}{(S^n+I_c^n)(1+h(\mu +d_c (\nu )+r_1))} \end{aligned}$$

We are concentrating on a model in a fuzzy environment of a specific group of people with a triangular membership function. We examine it for various levels of viruses.

- **Case 1.** If $\nu <\nu _m$, then $\beta _c(\nu )=0$ and the above system becomes
  49 $$\begin{aligned} S^{n+1}& = \frac{(S^n+\Lambda h+hr_1I_c^n)(S^n+I_c^n)}{S^n+I_c^n+h\mu (S^n+I_c^n)} \end{aligned}$$
  50 $$\begin{aligned} {I_c^{n+1}}& = \frac{I_c^n (S^n+I_c^n )}{(S^n+I_c^n)(1+h(\mu +d_c (\nu )+r_1))} \end{aligned}$$
- **Case 2.** If $\nu _m\le \nu \le \nu _0$, then $\beta _c(\nu )=\frac{\nu -\nu _m}{\nu _0-\nu _m}$ and the above system becomes
  51 $$\begin{aligned} S^{n+1}& = \frac{(S^n+\Lambda h+hr_1I_c^n)(S^n+I_c^n)}{S^n+I_c^n+h(\beta _c(\nu )I_c^n+\mu (S^n+I_c^n))} \end{aligned}$$
  52 $$\begin{aligned} {I_c^{n+1}}& = \frac{I_c^n (S^n+I_c^n )+\beta _c(\nu )I_c^n S^n}{(S^n+I_c^n)(1+h(\mu +d_c (\nu )+r_1))} \end{aligned}$$
- **Case 3.** If $\nu _0<\nu <\nu _M$, then $\beta _c(\nu )=1$ and the above system becomes
  53 $$\begin{aligned} S^{n+1}& = \frac{(S^n+\Lambda h+hr_1I_c^n)(S^n+I_c^n)}{S^n+I_c^n+h(I_c^n+\mu (S^n+I_c^n))} \end{aligned}$$
  54 $$\begin{aligned} {I_c^{n+1}}& = \frac{I_c^n (S^n+I_c^n )+I_c^n S^n}{(S^n+I_c^n)(1+h(\mu +d_c (\nu )+r_1))} \end{aligned}$$

### Convergence analysis of the NSFD scheme

Convergence analysis examines whether the numerical solution obtained by a numerical method approaches the true solution of the underlying mathematical problem. The eigenvalues of the Jacobean matrix at an equilibrium point are important in determining the system’s convergence behavior. If all of the eigenvalues of the Jacobean have magnitudes strictly less than one, the system’s trajectories will converge towards the equilibrium point over time. If any eigenvalue has a magnitude greater than one, the related trajectories will diverge from the equilibrium point. In such instances, the system will fail to converge to equilibrium, and the behavior may become chaotic or unpredictable. In this part, we will discuss the convergence of the NSFD scheme for the above model. The system (47, 48) can be written as

55 $$\begin{aligned} A_7& = \frac{(S^n+\Lambda h+hr_1I_c^n)(S^n+I_c^n)}{S^n+I_c^n+h(\beta _c(\nu )I_c^n+\mu (S^n+I_c^n))} \end{aligned}$$

56 $$\begin{aligned} A_8& = \frac{I_c^n (S^n+I_c^n )+\beta _c(\nu )I_c^n S^n}{(S^n+I_c^n)(1+h(\mu +d_c (\nu )+r_1))} \end{aligned}$$

Jacobean of the system (55, 56) is

$$\begin{aligned} J=\left[ \begin{array}{cc} \frac{\partial A_7}{\partial S} &{} \frac{\partial A_7}{\partial I_c} \\ \frac{\partial A_8}{\partial S} &{} \frac{\partial A_8}{\partial I_c} \\ \end{array} \right] \end{aligned}$$

**Figure 2:**
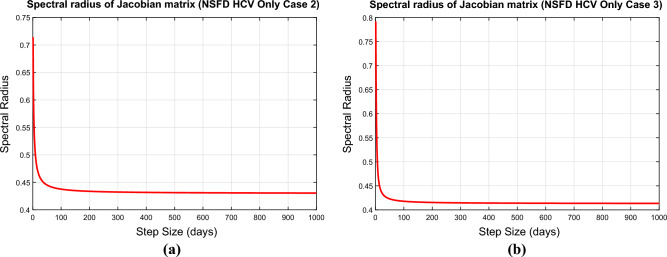
Eigen values of the Jacobean matrices corresponding to HCV only sub model (**a**) at the EE point (Case 2) (**b**) at the EE point (Case 3).

- **Case 1.** If $\nu <\nu _m$, then $\beta _c(\nu )=0$ and the above Jacobean matrix becomes
  $$\begin{aligned} J(E_h^0)=\left[ \begin{array}{cc} \frac{1}{1+h\mu } &{} \frac{hr_1}{1+h\mu } \\ 0&{} \frac{1}{1+h(\mu +d_c (\nu )+r_1 )} \\ \end{array} \right] \end{aligned}$$
  Here $\lambda _1=\frac{hr_1}{1+h\mu }$ and $\lambda _2=\frac{1}{1+h(\mu +d_c (\nu )+r_1 )}$. Since both eigenvalues of the above Jacobean matrix are less than 1, therefore, the proposed scheme is unconditionally convergent.
- **Case 2.** If $\nu _m\le \nu \le \nu _0$, then $\beta _c(\nu )=\frac{\nu -\nu _m}{\nu _0-\nu _m}$ and the Jacobean of the system (55, 56) becomes
  $$\begin{aligned} J(E_h^*)=\left[ \begin{array}{cc} \frac{\partial A_7}{\partial S}(E_h^*) &{} \frac{\partial A_7}{\partial I_c}(E_h^*) \\ \frac{\partial A_8}{\partial S}(E_h^*) &{} \frac{\partial A_8}{\partial I_c}(E_h^*) \\ \end{array} \right] \end{aligned}$$
  where
  $$\begin{aligned}{} & {} \frac{\partial A_7}{\partial S}(E_h^*) =\frac{(S^*+I_c^*)^2+h(S^*+I_c^* )(\beta _c(\nu )I_c^*+\mu (S^*+I_c^* ))+h\beta _c(\nu )I_c^* (S^*+\Lambda h+hr_1 I_c^*)}{[S^*+I_c^*+h(\beta _c(\nu )I_c^*+\mu (S^*+I_c^* ))]^2},\\{} & {} \frac{\partial A_7}{\partial I_c}(E_h^*)=\frac{hr_1 (S^*+I_c^* )^2+h^2 r_1 (S^*+I_c^* )(\beta _c(\nu )I_c^*+\mu (S^*+I_c^* ))-h\beta _c(\nu )S^* (S^*+\Lambda h+hr_1 I_c^* )}{[S^*+I_c^*+h(\beta _c(\nu )I_c^*+\mu (S^*+I_c^* ))]^2},\\{} & {} \frac{\partial A_8}{\partial S}(E_h^*)=\frac{h\beta _c(\nu ){I_c^*}^2 (1+h(\mu +d_c (\nu )+r_1 ))}{[(S^*+I_c^* )(1+h(\mu +d_c (\nu )+r_1 ))]^2} \end{aligned}$$
  and
  $$\begin{aligned} \frac{\partial A_8}{\partial I_c}(E_h^*)=\frac{(S^*+I_c^* )^2 (1+h(\mu +d_c (\nu )+r_1 ))+h\beta _c(\nu ) {S^*}^2 (1+h(\mu +d_c (\nu )+r_1 ))}{[(S^*+I_c^* )(1+h(\mu +d_c (\nu )+r_1 ))]^2 } \end{aligned}$$
- **Case 3.** If $\nu _0<\nu <\nu _M$, then $\beta _c(\nu )=1$ and the Jacobean of the system (55, 56) becomes
  $$\begin{aligned} J(E_h^{**})=\left[ \begin{array}{cc} \frac{\partial A_7}{\partial S}(E_h^{**}) &{} \frac{\partial A_7}{\partial I_c}(E_h^{**}) \\ \frac{\partial A_8}{\partial S}(E_h^{**}) &{} \frac{\partial A_8}{\partial I_c}(E_h^{**}) \\ \end{array} \right] \end{aligned}$$
  where
  $$\begin{aligned}{} & {} \frac{\partial A_7}{\partial S} =\frac{(S^{**}+I_c^{**})^2+h(S^{**}+I_c^{**} )(I_c^{**}+\mu (S^{**}+I_c^{**} ))+hI_c^{**} (S^{**}+\Lambda h+hr_1 I_c^{**})}{[S^{**}+I_c^{**}+h(I_c^{**}+\mu (S^{**}+I_c^{**} ))]^2},\\{} & {} \frac{\partial A_7}{\partial I_c}=\frac{hr_1 (S^{**}+I_c^{**} )^2+h^2 r_1 (S^{**}+I_c^{**} )(I_c^{**}+\mu (S^{**}+I_c^{**} ))-hS^{**} (S^{**}+\Lambda h+hr_1 I_c^{**} )}{[S^{**}+I_c^{**}+h(I_c^{**}+\mu (S^{**}+I_c^{**} ))]^2},\\{} & {} \frac{\partial A_8}{\partial S}=\frac{(h{I_c^{**}}^2 (1+h(\mu +d_c (\nu )+r_1 ))}{[(S^{**}+I_c^{**} )(1+h(\mu +d_c (\nu )+r_1 ))]^2} \end{aligned}$$
  and
  $$\begin{aligned} \frac{\partial A_8}{\partial I_c}=\frac{(S^{**}+I_c^{**})^2 (1+h(\mu +d_c (\nu )+r_1 ))+h {S^{**}}^2 (1+h(\mu +d_c (\nu )+r_1 ))}{[(S^{**}+I_c^{**} )(1+h(\mu +d_c (\nu )+r_1 ))]^2 } \end{aligned}$$
  The MATLAB database was used to plot the principal eigenvalues of the Jacobeans $J_1$ and $J_2$, which are shown in Fig. 2a and b. The fact that all of these greatest eigenvalues are less than one verifies the intended assertion.

### NSFD scheme for HIV only sub model

NSFD scheme for the system (35–38) is

57 $$\begin{aligned} S^{n+1}& = \frac{(S^n+h\Lambda )(S^n+I_h^n+A_h^n+A_t^n)}{(S^n+I_h^n+A_h^n+A_t^n )+h(\mu (S^n+I_h^n+A_h^n+A_t^n )+\beta _h(\nu )I_h^n)} \end{aligned}$$

58 $$\begin{aligned} I_h^{n+1}& = \frac{I_h^n [(S^n+I_h^n+A_h^n+A_t^n )+h\beta _h(\nu ) S^n)]}{(S^n+I_h^n+A_h^n+A_t^n )(1+h(\mu +\rho _1 )) } \end{aligned}$$

59 $$\begin{aligned} A_h^{n+1}& = \frac{A_h^n+h\rho _1 I_h^n}{1+h(\mu +d_a (\nu )+\theta _1)} \end{aligned}$$

60 $$\begin{aligned} A_t^{n+1}& = \frac{A_t^n+h\theta _1 A_h^n}{1+h(\mu +d_a (\nu ))} \end{aligned}$$

Again, we investigate the model across varying viral quantities, given our concentration on a specific demographic within a fuzzy context through a triangular membership function.

- **Case 1.** If $\nu <\nu _m$, then $\beta _h(\nu )=0$ and the above system becomes
  61 $$\begin{aligned} S^{n+1}& = \frac{(S^n+h\Lambda )(S^n+I_h^n+A_h^n+A_t^n)}{(S^n+I_h^n+A_h^n+A_t^n )+h(\mu (S^n+I_h^n+A_h^n+A_t^n )} \end{aligned}$$
  62 $$\begin{aligned} I_h^{n+1}& = \frac{I_h^n (S^n+I_h^n+A_h^n+A_t^n )}{(S^n+I_h^n+A_h^n+A_t^n )(1+h(\mu +\rho _1 )) } \end{aligned}$$
  63 $$\begin{aligned} A_h^{n+1}& = \frac{A_h^n+h\rho _1 I_h^n}{1+h(\mu +d_a (\nu )+\theta _1)} \end{aligned}$$
  64 $$\begin{aligned} A_t^{n+1} &= \frac{A_t^n+h\theta _1 A_h^n}{1+h(\mu +d_a (\nu ))} \end{aligned}$$
- **Case 2.** If $\nu _m\le \nu \le \nu _0$, then $\beta _h(\nu )=\frac{\nu -\nu _m}{\nu _0-\nu _m}$ and the above system becomes
  65 $$\begin{aligned} S^{n+1}& = \frac{(S^n+h\Lambda )(S^n+I_h^n+A_h^n+A_t^n)}{(S^n+I_h^n+A_h^n+A_t^n )+h(\mu (S^n+I_h^n+A_h^n+A_t^n )+\beta _h(\nu )I_h^n)} \end{aligned}$$
  66 $$\begin{aligned} I_h^{n+1}& = \frac{I_h^n [(S^n+I_h^n+A_h^n+A_t^n )+h\beta _h(\nu ) S^n)]}{(S^n+I_h^n+A_h^n+A_t^n )(1+h(\mu +\rho _1 )) } \end{aligned}$$
  67 $$\begin{aligned} A_h^{n+1}& = \frac{A_h^n+h\rho _1 I_h^n}{1+h(\mu +d_a (\nu )+\theta _1)} \end{aligned}$$
  68 $$\begin{aligned} A_t^{n+1}& = \frac{A_t^n+h\theta _1 A_h^n}{1+h(\mu +d_a (\nu ))} \end{aligned}$$
- **Case 3.** If $\nu _0<\nu <\nu _M$, then $\beta _h(\nu )=1$ and the above system becomes
  69 $$\begin{aligned} S^{n+1}& = \frac{(S^n+h\Lambda )(S^n+I_h^n+A_h^n+A_t^n)}{(S^n+I_h^n+A_h^n+A_t^n )+h(\mu (S^n+I_h^n+A_h^n+A_t^n ))} \end{aligned}$$
  70 $$\begin{aligned} I_h^{n+1}& = \frac{I_h^n (S^n+I_h^n+A_h^n+A_t^n )}{(S^n+I_h^n+A_h^n+A_t^n )(1+h(\mu +\rho _1 )) } \end{aligned}$$
  71 $$\begin{aligned} A_h^{n+1}& = \frac{A_h^n+h\rho _1 I_h^n}{1+h(\mu +d_a (\nu )+\theta _1)} \end{aligned}$$
  72 $$\begin{aligned} A_t^{n+1}& = \frac{A_t^n+h\theta _1 A_h^n}{1+h(\mu +d_a (\nu ))} \end{aligned}$$

### Convergence analysis of NSFD scheme

In this part, we will discuss the convergence of NSFD scheme for the above model. The system (57)–(60) can be written as

73 $$\begin{aligned} K& = \frac{(S^n+h\Lambda )(S^n+I_h^n+A_h^n+A_t^n)}{(S^n+I_h^n+A_h^n+A_t^n )+h(\mu (S^n+I_h^n+A_h^n+A_t^n )+\beta _h(\nu )I_h^n)} \end{aligned}$$

74 $$\begin{aligned} L& = \frac{I_h^n [(S^n+I_h^n+A_h^n+A_t^n )+h\beta _h(\nu ) S^n)]}{(S^n+I_h^n+A_h^n+A_t^n )(1+h(\mu +\rho _1 )) } \end{aligned}$$

75 $$\begin{aligned} M& = \frac{A_h^n+h\rho _1 I_h^n}{1+h(\mu +d_a (\nu )+\theta _1)} \end{aligned}$$

76 $$\begin{aligned} N& = \frac{A_t^n+h\theta _1 A_h^n}{1+h(\mu +d_a (\nu ))} \end{aligned}$$

The Jacobean matrix corresponding to (73)–(76) is

$$\begin{aligned} J=\left[ \begin{array}{cccc} \frac{\partial K}{\partial S} &{} \frac{\partial K}{\partial I_h} &{} \frac{\partial K}{\partial A_h}&{} \frac{\partial K}{\partial A_t} \\ \frac{\partial L}{\partial S} &{} \frac{\partial L}{\partial I_h} &{} \frac{\partial L}{\partial A_h}&{} \frac{\partial L}{\partial A_t} \\ \frac{\partial M}{\partial S} &{} \frac{\partial M}{\partial I_h} &{} \frac{\partial M}{\partial A_h} &{} \frac{\partial M}{\partial A_t} \\ \frac{\partial N}{\partial S} &{} \frac{\partial N}{\partial I_h} &{} \frac{\partial N}{\partial A_h} &{} \frac{\partial N}{\partial A_t} \\ \end{array} \right] \end{aligned}$$

**Figure 3:**
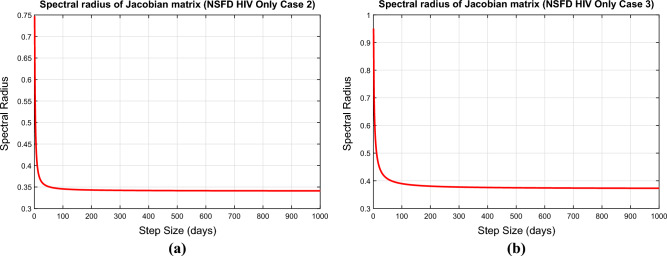
Eigen values of the Jacobean matrices corresponding to HIV only sub model (**a**) at the EE point (Case 2) (**b**) at the EE point (Case 3).

- **Case 1.** The above Jacobean matrix at VFE is
  $$\begin{aligned} J=\left[ \begin{array}{cccc} \frac{1}{1+h\mu } &{} 0&{} 0&{} 0 \\ 0 &{} \frac{1}{1+h(\mu +\rho _1)}&{} 0&{} 0 \\ 0 &{}\frac{h\rho _1}{1+h(\mu +d_a (\nu )+\theta _1)} &{} \frac{1}{1+h(\mu +d_a (\nu )+\theta _1)} &{} 0 \\ 0 &{} 0 &{} \frac{1}{1+h(\mu +d_a (\nu )+\theta _1)} &{}\frac{1}{1+h(\mu +d_a (\nu ))}\\ \end{array} \right] \end{aligned}$$
  From the above Jacobean matrix we obtain the eigenvalue $\lambda _1=\frac{1}{1+h\mu }<1$, $\lambda _2=\frac{1}{1+h(\mu +\rho _1)}<1$, $\lambda _3=\frac{1}{1+h(\mu +d_a (\nu )+\theta _1)}<1$ and $\lambda _4=\frac{1}{1+h(\mu +d_a (\nu ))}<1$. Since all the eigenvalues of Jacobean at VFE are less than one, therefore, the proposed numerical scheme will converge to VFE if $R_h<1$ irrespective of the step size taken. Hence the VFE is stable if $R_h<1$.
- **Case 2.** The Jacobean matrix corresponding to case 2 can be written as
  $$\begin{aligned} J(E_h^*)=\left[ \begin{array}{cccc} \frac{\partial K}{\partial S}(E_h^*) &{} \frac{\partial K}{\partial I_h}(E_h^*) &{} \frac{\partial K}{\partial A_h}(E_h^*)&{} \frac{\partial K}{\partial A_t}(E_h^*) \\ \frac{\partial L}{\partial S}(E_h^*) &{} \frac{\partial L}{\partial I_h}(E_h^*) &{} \frac{\partial L}{\partial A_h}(E_h^*)&{} \frac{\partial L}{\partial A_t}(E_h^*) \\ \frac{\partial M}{\partial S}(E_h^*) &{} \frac{\partial M}{\partial I_h}(E_h^*) &{} \frac{\partial M}{\partial A_h}(E_h^*) &{} \frac{\partial M}{\partial A_t}(E_h^*) \\ \frac{\partial N}{\partial S}(E_h^*) &{} \frac{\partial N}{\partial I_h}(E_h^*) &{} \frac{\partial N}{\partial A_h} (E_h^*)&{} \frac{\partial N}{\partial A_t}(E_h^*) \\ \end{array} \right] \end{aligned}$$
  where
  $$\begin{aligned} \frac{\partial K}{\partial S}(E_h^*)& = \frac{(S+I_h+A_h+A_t )^2 (1+h\mu )+h\beta _h (\nu )I_h [(S+\Lambda h)+(S+I_h+A_h+A_t )]}{[(S+I_h+A_h+A_t )+h(\mu (S+I_h+A_h+A_t )+\beta _h (\nu )I_h )]^2 },\\ \frac{\partial K}{\partial I_h}(E_h^*)& = \frac{-h\beta _h (\nu )(S+\Lambda h)(S+A_h+A_t )}{[(S+I_h+A_h+A_t )+h(\mu (S+I_h+A_h+A_t )+\beta _h (\nu )I_h )]^2 },\\ \frac{\partial K}{\partial A_h}(E_h^*)& = \frac{h\beta _h (\nu )I_h (S+\Lambda h)}{[(S+I_h+A_h+A_t )+h(\mu (S+I_h+A_h+A_t )+\beta _h (\nu )I_h )]^2 },\\ \frac{\partial K}{\partial A_t}(E_h^*)& = \frac{h\beta _h (\nu )I_h (S+\Lambda h)}{[(S+I_h+A_h+A_t )+h(\mu (S+I_h+A_h+A_t )+\beta _h (\nu )I_h )]^2 },\\ \frac{\partial L}{\partial S}(E_h^*)& = \frac{h\beta _h (\nu )I_h (I_h+A_h+A_t )(1+h(\mu +\rho _1 ))}{[(S+I_h+A_h+A_t )(1+h(\mu +\rho _1 ))]^2 },\\ \frac{\partial L}{\partial I_h}(E_h^*)& = \frac{(S+I_h+A_h+A_t )^2 (1+h(\mu +\rho _1 ))+h\beta _h (\nu )S(S+A_h+A_t )(1+h(\mu +\rho _1 ))}{[(S+I_h+A_h+A_t )(1+h(\mu +\rho _1 ))]^2},\\ \frac{\partial L}{\partial A_h}(E_h^*)& = \frac{-h\beta _h (\nu )I_h S(1+h(\mu +\rho _1))}{ [(S+I_h+A_h+A_t )(1+h(\mu +\rho _1))]^2 },\\ \frac{\partial L}{\partial A_t}(E_h^*)& = \frac{-h\beta _h (\nu )I_h S(1+h(\mu +\rho _1))}{[(S+I_h+A_h+A_t )(1+h(\mu +\rho _1))]^2},\\ \frac{\partial M}{\partial S}(E_h^*)& = 0,\\ \frac{\partial M}{\partial I_h}(E_h^*)& = \frac{h\rho _1}{1+h(\mu +d_a (\nu )+\theta _1)},\\ \frac{\partial M}{\partial A_h}(E_h^*)& = 0,\\ \frac{\partial M}{\partial A_t}(E_h^*)& = \frac{1}{1+h(\mu +d_a (\mu )+\theta _1)},\\ \frac{\partial N}{\partial S}(E_h^*)& = 0,\\ \frac{\partial N}{\partial I_h}(E_h^*)& = 0,\\ \frac{\partial N}{\partial A_h}(E_h^*)& = \frac{h\theta _1}{1+h(\mu +d_a (\nu ))},\\ \frac{\partial N}{\partial A_t}(E_h^*)& = \frac{1}{1+h(\mu +d_a (\nu ))}, \end{aligned}$$
- **Case 3.** The Jacobean matrix corresponding to case 3 can be written as
  $$\begin{aligned} J(E_h^{**})=\left[ \begin{array}{cccc} \frac{\partial K}{\partial S}(E_h^{**}) &{} \frac{\partial K}{\partial I_h}(E_h^{**}) &{} \frac{\partial K}{\partial A_h}(E_h^{**})&{} \frac{\partial K}{\partial A_t}(E_h^{**}) \\ \frac{\partial L}{\partial S}(E_h^{**}) &{} \frac{\partial L}{\partial I_h}(E_h^{**}) &{} \frac{\partial L}{\partial A_h}(E_h^{**})&{} \frac{\partial L}{\partial A_t}(E_h^{**}) \\ \frac{\partial M}{\partial S}(E_h^{**}) &{} \frac{\partial M}{\partial I_h}(E_h^{**}) &{} \frac{\partial M}{\partial A_h}(E_h^{**}) &{} \frac{\partial M}{\partial A_t}(E_h^{**}) \\ \frac{\partial N}{\partial S}(E_h^{**}) &{} \frac{\partial N}{\partial I_h}(E_h^{**}) &{} \frac{\partial N}{\partial A_h} (E_h^{**})&{} \frac{\partial N}{\partial A_t}(E_h^{**}) \\ \end{array} \right] \end{aligned}$$
  where
  $$\begin{aligned} \frac{\partial K}{\partial S}(E_h^{**})& = \frac{(S+I_h+A_h+A_t )^2 (1+h\mu )+hI_h [(S+\Lambda h)+(S+I_h+A_h+A_t )]}{[(S+I_h+A_h+A_t )+h(\mu (S+I_h+A_h+A_t )+I_h )]^2 },\\ \frac{\partial K}{\partial I_h}(E_h^{**})& = \frac{-h(S+\Lambda h)(S+A_h+A_t )}{[(S+I_h+A_h+A_t )+h(\mu (S+I_h+A_h+A_t )+I_h )]^2 },\\ \frac{\partial K}{\partial A_h}(E_h^{**})& = \frac{hI_h (S+\Lambda h)}{[(S+I_h+A_h+A_t )+h(\mu (S+I_h+A_h+A_t )+I_h )]^2},\\ \frac{\partial K}{\partial A_t}(E_h^{**})& = \frac{hI_h (S+\Lambda h)}{[(S+I_h+A_h+A_t )+h(\mu (S+I_h+A_h+A_t )+I_h )]^2 },\\ \frac{\partial L}{\partial S}(E_h^{**})& = \frac{hI_h (I_h+A_h+A_t )(1+h(\mu +\rho _1))}{[(S+I_h+A_h+A_t )(1+h(\mu +\rho _1 ))]^2},\\ \frac{\partial L}{\partial I_h}(E_h^{**})& = \frac{(S+I_h+A_h+A_t )^2 (1+h(\mu +\rho _1 ))+hS(S+A_h+A_t )(1+h(\mu +\rho _1 ))}{[(S+I_h+A_h+A_t )(1+h(\mu +\rho _1 ))]^2},\\ \frac{\partial L}{\partial A_h}(E_h^{**})& = \frac{-hI_h S(1+h(\mu +\rho _1))}{ [(S+I_h+A_h+A_t )(1+h(\mu +\rho _1))]^2 },\\ \frac{\partial L}{\partial A_t}(E_h^{**})& = \frac{-hI_h S(1+h(\mu +\rho _1))}{[(S+I_h+A_h+A_t )(1+h(\mu +\rho _1))]^2},\\ \frac{\partial M}{\partial S}(E_h^{**})& = 0,\\ \frac{\partial M}{\partial I_h}(E_h^{**})& = \frac{h\rho _1}{1+h(\mu +d_a (\nu )+\theta _1)},\\ \frac{\partial M}{\partial A_h}(E_h^{**})& = 0,\\ \frac{\partial M}{\partial A_t}(E_h^{**})& = \frac{1}{1+h(\mu +d_a (\mu )+\theta _1)},\\ \frac{\partial N}{\partial S}(E_h^{**})& = 0,\\ \frac{\partial N}{\partial I_h}(E_h^{**})& = 0,\\ \frac{\partial N}{\partial A_h}(E_h^{**})& = \frac{h\theta _1}{1+h(\mu +d_a (\nu ))},\\ \frac{\partial N}{\partial A_t}(E_h^{**})& = \frac{1}{1+h(\mu +d_a (\nu ))}, \end{aligned}$$
  The principal eigenvalues for the Jacobians $$J(E_h^{*})$$ and $$J(E_h^{**})$$ were plotted using the MATLAB database and are shown in Fig. 3a and b, respectively. The anticipated claim is supported by the finding that these largest eigenvalues are all less than unity.

Before closing this section, we provide some numerical simulations for the co-infection model of HIV/AIDS and Hepatitis C viruses with fuzzy parameters.

**Figure 4:**
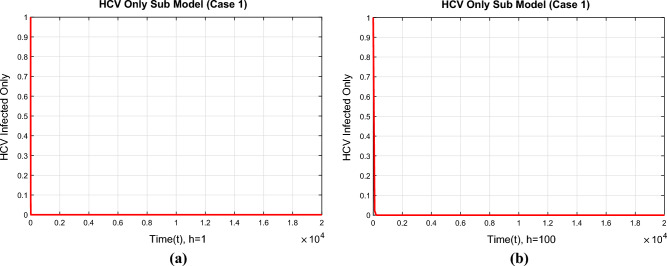
The portions of HCV infected population for case 1 (**a**) at $h=1$ (**b**) $h=100$.

Figure 4a,b show the convergence of the NSFD method to the true equilibrium points of the continuous model at step sizes, $h=1$ and $h=100$ at the first endemic equilibrium point. The figures show that the NSFD method remains convergent and retains the essential properties of the continuous dynamical system like positivity and boundedness.

**Figure 5:**
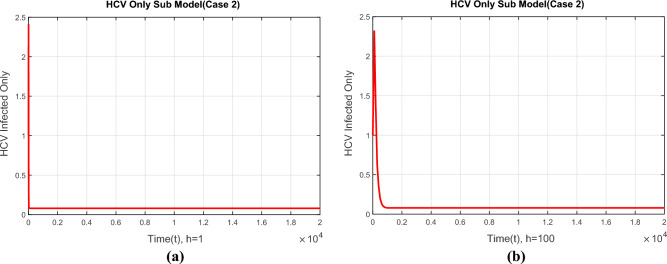
The portions of HCV infected population for case 2 (**a**) at $h=1$ (**b**) $h=100$.

The compartment of the HCV-infected subpopulation at the first EE point for case 2 is shown in Fig. 5a,b using the suggested NSFD approach with step sizes of $h=1$ and $h=100$. The demonstrated results highlight the method’s positive behavior and convergence. Due to this result, we may conclude that the suggested technique successfully represents the true dynamics of the disease at the first EE point.

**Figure 6:**
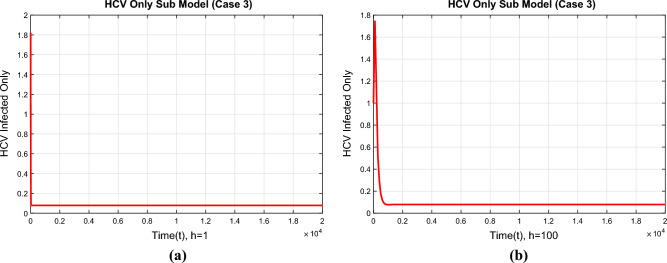
The portions of HCV infected population for case 3 (**a**) at $h=1$ (**b**) $h=100$.

Figure 6a,b show the compartment of the subpopulation HCV infected population at step sizes $h=1$ and $h=100$ respectively at the second EE point. The developed NSFD method converges to the true equilibrium points of the continuous model at different step sizes. This shows that the NSFD method preserves all the essential properties of the continuous dynamical system for case 3.

**Figure 7:**
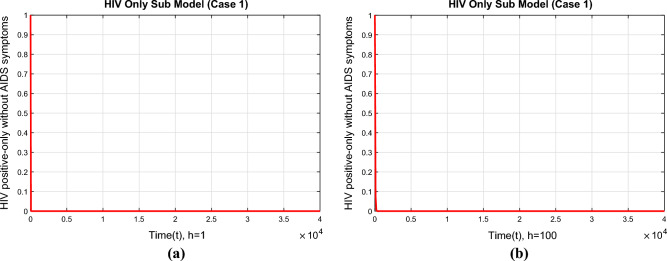
The portions of HIV infected population for case 1 (**a**) at $h=1$ (**b**) $h=100$.

Figure 7 shows how the compartment representing the HIV-positive group converges at the DFE point. The shown outcomes demonstrate the convergence of the NSFD technique as the time step increases to $h=1$ and $h=100$, while maintaining the significant properties of the continuous dynamical system.

**Figure 8:**
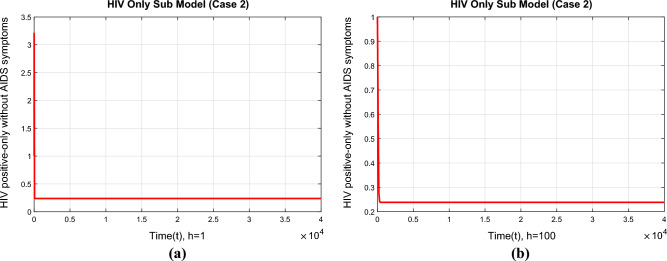
The portions of HIV infected population for case 2 (**a**) at $h=1$ (**b**) $h=100$.

Figure 8a and b show the compartment indicating the HIV-infected subpopulation’s positivity and convergence at the initial EE point. The graphs demonstrate how the NSFD technique effectively retains all of the fundamental properties of the continuous dynamical system when the time step grows to $h=1$ and $h=100$.

**Figure 9:**
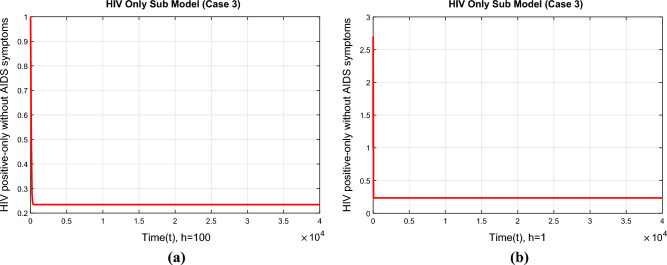
The portions of HIV infected population for case 3 (**a**) at $h=1$ (**b**) $h=100$.

In Fig. 9a and b, the behavior of the compartment of the subpopulation HIV infected population at step sizes $h=1$ and $h=100$ respectively for the second EE point have been shown. The figures show the positivity and convergence of the method again. In epidemic models, convergence and positivity are crucial characteristics. Evidently, the presented graphs show that for all equilibrium points, the proposed NSFD approach achieves convergence towards the real equilibrium points throughout a range of step sizes. This demonstrates that the approach is an effective tool for studying the model’s long-term behavior. We also determined that the method is suited for studying disease dynamics epidemic models based on this behavior. It can also be concluded from this behavior that the NSFD technique is capable of reflecting the dynamics of the studied model under fuzzy settings. Some conventional standard techniques that exist in the literature can produce chaos and misleading variations for particular discretization constraints^37, 38^.

## Conclusion

HIV and HCV co-infection presents a significant medical problem. Both viruses can be spread via common methods, such as unprotected sex and needle sharing, rendering particular populations more vulnerable. When a person is infected with both viruses, the interactions between them might hasten illness progression and complicate therapy. HIV affects the immune system, making infection resistance more difficult. HCV predominantly affects the liver and has the potential to cause chronic liver disease. When both viruses are present, their effects can amplify. Coinfection frequently accelerates the progression of liver disease, increases the risk of opportunistic infections, and increases the possibility of serious consequences. Mathematical modeling is critical in understanding and managing HIV and HCV coinfection. Fuzzy parameters play an important role in mathematical models, particularly in situations where there is uncertainty or imprecision in the data or parameters used in the model. It allows for a more realistic representation of real-world scenarios and aids in making well-informed decisions based on a range of potential outcomes. In this research, we have explored a co-infection model involving HIV/AIDS and Hepatitis C virus, incorporating fuzzy parameters. Our approach considers the uneven transmission of the diseases among infected individuals, where the level of disease transmission varies based on the individual’s virus quantity. Likewise, the disease-induced mortality differ among individuals. Rather, they differ among each individual within the population. This is where the introduction of the fuzzy model brings a notable advantage, offering a more adaptable and well-balanced perspective compared to the conventional crisp system. The incorporation of fuzzy theory proves valuable in addressing uncertainties inherent in mathematical disease modeling. Fuzzy variables, being contingent on virus load linked to the viral quantities, are examined across varying viral levels. With this in mind, we investigated the fuzzy equilibrium points of the analyzed model while accounting for virus amount in the population. Our findings revealed that the VFE point is reached when the viral abundance in the population remains below the threshold required for disease transmission. However, as virus load over the lowest thresholds required for transmission, the EE points are reached. We estimated the basic reproduction number and studied its variations with different viral amounts using next-generation matrix methods, giving the fuzzy basic reproduction number.

Furthermore, we have formulated an NSFD scheme applicable to both the HIV-Only Sub model and the HCV Only Sub Model. We subjected these schemes to analysis across varying viral load levels. Given that ensuring the positivity of solutions in dynamic population models is a primary objective, the introduced numerical technique maintains this positivity, as demonstrated in this article. The resilience of the devised approach is demonstrated by the fact that it constantly maintains positivity not just for different viral amounts but also when dealing with both small and large step sizes. Another critical attribute for dynamic population models is convergence. In this regard, the developed scheme maintains convergence at both the VFE and EE points. In the future, this study could be expanded in a variety of ways, including combining fuzzy stochastic, fuzzy delayed, and fuzzy fractional dynamic elements, as well as considering saturated incidence, treatment effects, and delays with fuzzy parameters. Furthermore, the NSFD modeling theory might be extended to include age-structured fuzzy epidemic models and investigate a variety of other possibilities.

## Data Availability

The datasets analyzed during the current study are available from the first author upon reasonable request.
